# *BMC Psychiatry* reviewer acknowledgement 2015

**DOI:** 10.1186/s12888-016-0738-4

**Published:** 2016-02-09

**Authors:** Anna Clark

**Affiliations:** BioMed Central, Floor 6, 236 Gray’s Inn Road, London, WC1X 8HB UK

## Abstract

The editors of *BMC Psychiatry* would like to thank all our reviewers who have contributed to the journal in Volume 15 (2015).

**Melanie Abas**

UK

**Allan Abbass**

Canada

**Giovanni Abbate-Daga**

Italy

**Illiam Adams**

USA

**Arpana Agrawal**

USA

**Sari Ahlqvist-Björkroth**

Finland

**Rayan Al Jurdi**

USA

**Steven Albert**

USA

**Chelsea Ale**

USA

**Olutayo Aloba**

Nigeria

**Kursat Altinbas**

Turkey

**Shiri Altman**

Israel

**Mario Amore**

Italy

**Lars P. H. Andersen**

Denmark

**Kelly Anderson**

Canada

**Janis Anderson**

USA

**Paul Andrews**

Canada

**Michael Anestis**

USA

**Tokie Anme**

Japan

**N. N. Ansari**

Iran

**Daniel Antonius**

USA

**Niki Antypa**

Italy

**Gregory Armstrong**

Australia

**Danilo Arnone**

UK

**Jafet Arrieta**

USA

**Ryosuke Asano**

Japan

**Philip J. Asherson**

UK

**Majed Ashy**

USA

**Anu Asnaani**

USA

**Leslie Atkinson**

Canada

**Mareike Augsburger**

Germany

**Dara Babinski**

USA

**Shannon Bader**

USA

**Cristiane Baes**

Brazil

**Muideen Bakare**

Nigeria

**Jess Baker**

Australia

**Dinarte Ballester**

Brazil

**Ryan Balzan**

Australia

**Rebecca Bargenquast**

Australia

**Antonia Barke**

Germany

**Guy Barry**

Australia

**Cali Bartholomeusz**

Australia

**S. Barzilay**

Israel

**Madhur Basnet**

Nepal

**Ignacio Basurte-Villamor**

Spain

**Annette Bauer**

UK

**Guy Beauchamp**

Canada

**Chalotte Beaudart**

Belgium

**Margherita Bechi**

Italy

**Thomas Becker**

Germany

**Giorgio Bedogni**

Italy

**Jeffrey Bedwell**

Germany

**Martino Belvederi Murri**

Italy

**Kelly Benke**

USA

**Justin Benzer**

USA

**Michael Bergin**

Ireland

**Matthias Berking**

Germany

**Gail Bernstein**

USA

**Fabrizio Bert**

Italy

**Chris Bervoets**

Belgium

**Arvin Bhana**

USA

**M.S. Bhatia**

India

**Joanna Biernacka**

USA

**Victoria Bird**

UK

**Rachel Birnbaum**

Canada

**Dorothy Bishop**

UK

**Jeffrey Bishop**

USA

**Tonmoy Biswas**

Bangladesh

**Erla Bjornsdottir**

Iceland

**Emma Blackmore**

USA

**Simon Blackwell**

UK

**Sergio Blay**

Brazil

**lse Blignault**

Australia

**Michael Bloch**

USA

**Victor Blueml**

Austria

**Mitja Bodatsch**

Germany

**Katja Boehm**

Germany

**Yvonne Bohr**

UK

**Maria Clara Bonaglia**

Italy

**Kirsten Bond**

UK

**Gary Bond**

USA

**Mark Boschen**

Australia

**Michelle Bosquet Enlow**

USA

**Maria Böttche**

Germany

**Rami Bou Khalil**

Lebanon

**Philip Boyce**

Australia

**Nicole Boyer**

UK

**Laura Boylan**

USA

**Eva-Lotta Brakemeier**

Germany

**Jorgen Bramness**

Norway

**Mary-Lynn Brecht**

USA

**Benjamin Brent**

USA

**Joshua Breslau**

USA

**Christian Brettschneider**

Germany

**Francis Bridges**

USA

**Rob H. S. Van Den Brink**

The Netherlands

**Thomas Britt**

Vanuatu

**Marc Broadbent**

Australia

**Lisa Brophy**

Australia

**Felicity Brown**

USA

**Rhonda Brown**

USA

**Matthew Bruce**

UK

**Annette Brühl**

Switzerland

**Eric Brunet-Gouet**

France

**Benjamin Buck**

USA

**Kelly Buck**

USA

**Chiara Buizza**

Italy

**Kyle Burghardt**

USA

**Hira Burhan**

Pakistan

**Lisa Burke**

Australia

**Michelle Burns**

USA

**Kenneth Butler**

USA

**Nancy Byatt**

USA

**Linda Byrne**

Australia

**Jennifer C. Britton**

USA

**Herve Caci**

France

**Amaia Calderón-Larrañaga**

Spain

**Okan Caliyurt**

Turkey

**Dan Campbell**

USA

**Marco Canevelli**

Italy

**Bo Cao**

USA

**Rochelle Caplan**

USA

**Nicole Caporino**

USA

**R. Nicholas Carleton**

Canada

**Claudia Carmassi**

Italy

**Giuseppe Carra**

UK

**Normand Carrey**

Canada

**Matteo Cella**

UK

**Christine Cha**

USA

**Monique Chaaya**

Lebanon

**Anil Chacko**

USA

**Melanie Chalder**

UK

**Wing Chung Chang**

Hong Kong

**Fiona Charlson**

Australia

**Somnath Chatterji**

Switzerland

**Shu-Ping Chen**

Canada

**Lizhang Chen**

China

**Henian Chen**

USA

**Ying-Yeh Chen**

Taiwan

**lexander Chervyakov**

Russia

**Keely Cheslack-Postava**

USA

**Manoranjenni Chetty**

UK

**Karine Chevreul**

France

**Carolyn Chew-Graham**

UK

**Rie Chiba**

Japan

**Marco Chiesa**

UK

**Terence Ching**

Singapore

**Luigi Rocco Chiri**

Italy

**Brad Christian**

USA

**Brad Christian**

USA

**Dorte M. Christiansen**

Denmark

**David Christmas**

UK

**Gary Christopher**

UK

**Julie Chronister**

USA

**Kuo-Hsuan Chung**

Taiwan

**In Won Chung**

South Korea

**David Cicero**

USA

**Ayse Ciftci**

USA

**Crystal Clark**

USA

**Mary Clarke**

Ireland

**Lars Clemmensen**

Denmark

**Vanessa Cobham**

Australia

**David Cohen**

France

**Dan Cohen**

The Netherlands

**Judith Cohen**

USA

**Giuseppe Colella**

Italy

**Claudio Colonnese**

Italy

**G. Colville**

UK

**Stefano Comai**

Italy

**Charlotte Connor**

UK

**Daniela Conrad**

Germany

**Deidre A. Conroy**

USA

**Angus Cook**

Australia

**William Coryell**

USA

**Doina Cosman**

Germany

**David Cottrell**

UK

**Philippe Courtet**

France

**Nancy Covell**

USA

**Leam Craig**

UK

**Laurie Craigen**

USA

**Linda Craighead**

USA

**Mike Crawford**

UK

**Henk Cremers**

The Netherlands

**Paul Cromarty**

Australia

**Vanessa Cropley**

Australia

**Stephanie Crowley**

USA

**Gary Cuddeback**

USA

**Xu Cui**

Australia

**Jackie Curtis**

Australia

**Boldizsar Czeh**

Hungary

**Pal Czobor**

Hungary

**Federico D'Agata**

Italy

**Rada Dagher**

USA

**Jessica Daniels**

USA

**Alison Darcy**

USA

**Sabrina Darrow**

USA

**Sarah Dash**

Australia

**Jose De Leon**

USA

**Claire De Oliveira**

Canada

**Varuni De Silva**

Sri Lanka

**Mahshid Dehghan**

Canada

**Eberhard A. Deisenhammer**

Austria

**Ac Del Re**

UK

**Ryan Delapp**

USA

**Vida Demarin**

Croatia

**Caroline Demily**

France

**Paul Desan**

USA

**Sangeeta Dey**

New Zealand

**Laura Di Bona**

UK

**Martina Di Simplicio**

UK

**Karola Dillenburger**

UK

**Giancarlo Dimaggio**

Italy

**Konstantinos Dimitrelis**

UK

**Alexandra Dingemans**

The Netherlands

**Diana Dinitto**

USA

**Tom Dinzeo**

USA

**Russell Dlae**

Australia

**Karen Dobkins**

USA

**David Dodell-Feder**

USA

**Ana Flavia D'Oliveira**

Brazil

**elli Dominick**

USA

**llie Dommett**

UK

**Uta-Susan Donges**

Germany

**Vonetta Dotson**

USA

**Sarah Doucette**

Canada

**Stella Dracheva**

USA

**Aaron Drummond**

Australia

**Hellen Livia Drumond Marra**

Brazil

**Todd Duhamel**

Canada

**Markus Duncan**

Canada

**Boadie Dunlop**

USA

**Serdar Dursun**

Canada

**Joy Duxbury**

UK

**John Eastwood**

Australia

**David Daniel Ebert**

Germany

**Chad Ebesutani**

South Korea

**Cindy Eckart**

Germany

**David Eddie**

USA

**Dawn Edge**

UK

**Karen-Leigh Edward**

Australia

**Tracy Eells**

USA

**Caroline Eggerding**

USA

**Hannelore Ehrenreich**

Germany

**Mehmet Cagdas Eker**

Turkey

**Mona Eklund**

Sweden

**Elisabeth Ekstrand**

Sweden

**Thomas Elbert**

Germany

**Catherine Elliot**

New Zealand

**Ian Elliott**

USA

**Jerome Endrass**

Switzerland

**Amalia Enriquez-De-Salamanca**

Spain

**Lene Falgaard Eplov**

Denmark

**Steven Erickson**

USA

**Oluyomi Esan**

Nigeria

**Pascale Esch**

Luxembourg

**Randall Espinoza**

USA

**Elmar Etzersdorfer**

Germany

**Jonas Everaert**

Belgium

**Harris Eyre**

Australia

**Ariel Eytan**

Switzerland

**Luis Falcato**

Switzerland

**Jianqun Fang**

China

**David Farabee**

USA

**Ali Farhoudian**

Iran

**Aida Farreny**

Spain

**Samantha Farris**

USA

**Secondo Fassino**

Italy

**Todd Favorite**

USA

**Mina Fazel**

UK

**David Fedele**

USA

**Brisa S. Fernandes**

Australia

**Maria Isabel Fernández San Martín**

Spain

**Fernando Fernandez-Aranda**

Spain

**Julio Fernandez-Mendoza**

USA

**Tulin Fidan**

Turkey

**Jess Fiedorowicz**

USA

**Jose Fiks**

Brazil

**Sacha Filia**

Australia

**Michael Fingerhood**

USA

**David Fink**

USA

**Donna Marie Fick**

USA

**Mario Fioravanti**

Italy

**Ruth Firmin**

USA

**Gloria Fischer**

Germany

**Patrick Fisher**

Denmark

**Karen Fisher**

USA

**Amanda Fitzgerald**

Ireland

**Valentin Flaudias**

France

**Paul Flaxman**

UK

**Edna Foa**

USA

**Liz Forty**

UK

**James Foulds**

New Zealand

**Guido Frank**

USA

**Alexis Frazier-Wood**

USA

**Mark Freeston**

UK

**Gregory Fricchione**

USA

**John Fromson**

USA

**Kevin Fuji**

USA

**Daniel Fulford**

USA

**Isaac Galatzer-Levy**

USA

**Igor Galynker**

USA

**Jenny Gamble**

Australia

**Theresa Gannon**

UK

**David Gardner**

Canada

**Joshua Garfield**

Australia

**Annie Garner**

USA

**Maria Gaspar**

Portugal

**Tian Ge**

USA

**Pierre Geoffroy**

USA

**Adam Gerace**

Australia

**James Gerhart**

USA

**Domenico Giacco**

UK

**Vasudha Gidugu**

USA

**Katrin Giel**

Germany

**Jacek Gierus**

Poland

**Eva Gilboa-Schechtman**

Israel

**Kenneth Gill**

USA

**Steve Gillard**

UK

**Rich Gilman**

USA

**Ylva Ginsberg**

Sweden

**Brenda Gladstone**

Canada

**David Gleaves**

Australia

**Fernando Goes**

USA

**Thomas Goetz**

Germany

**Miriam Goldberg**

USA

**Emily Goldmann**

USA

**Fastone Goma**

Zambia

**Lizzette Gómez-De-Regil**

Mexico

**Ali Saffet Gonul**

Turkey

**Sarah Goodday**

Canada

**Laura Goodwin**

UK

**Srihari Gopal**

USA

**Rakesh Goyal**

USA

**Hermine Graham**

UK

**Carla Maria Gramaglia**

Italy

**Sarah Gray**

Canada

**Trisha Greenhalgh**

UK

**Tracy Greer**

USA

**Margaret Griffin**

USA

**T. K. Grimholt**

Norway

**Sandeep Grover**

India

**Fanglin Guan**

China

**Wenbin Guo**

China

**Emilio Gutierrez**

Spain

**David Haaga**

USA

**Kassahun Habtamu**

Ethiopia

**Gareth Hagger-Johnson**

UK

**Yang Haicheng**

China

**Helinä Hakko**

Finland

**Hamada Hamid**

USA

**Tae-Un Han**

USA

**Kirsten Hancock**

Australia

**Charlotte Hanlon**

Ethiopia

**Ben Hannigan**

UK

**Eilis Hannon**

UK

**Ihtsham Haq**

USA

**Susanne Harder**

Denmark

**Ashley Harrison**

USA

**Andrea Hartmann**

Germany

**Carol Harvey**

Australia

**Abd Hasan**

UK

**Leslie Hasche**

USA

**Aili Hanim Hashim**

Malaysia

**Kenji Hashimoto**

Japan

**Ryu-Ichiro Hashimoto**

Japan

**Stefanie Hassel**

UK

**Ilanit Hasson-Ohayon**

Israel

**Ann Haynos**

USA

**Alexandre Heeren**

USA

**Eva Heim**

Switzerland

**Irma Hein**

The Netherlands

**A. Helaly**

Egypt

**Craig Henderson**

USA

**Julie Henderson**

Australia

**Claire Henderson**

UK

**Michael Henry**

USA

**Stephan Heres**

Germany

**Ryan Herringa**

USA

**Morten Hesse**

Denmark

**Oystein Hetlevik**

Norway

**Diego Hidalgo-Mazzei**

Spain

**Stacy Hill**

USA

**Claudia Hilton**

USA

**Andreas Hinz**

Germany

**Tetsu Hirosawa**

Japan

**Sheilagh Hodgins**

Canada

**Louise Hoeffding**

Denmark

**Michael Höfler**

Germany

**Ryan Hong**

Singapore

**Lindsey Hopkins**

USA

**Samantha Horswill**

Canada

**Anders Hovland**

Norway

**Richard Howard**

UK

**Tasha R. Howe**

USA

**Juergen Hoyer**

Germany

**Yueqin Huang**

China

**Xiaojun Huang**

China

**Leon Hubbard**

UK

**Markus Huber**

Italy

**Anita Hubley**

Canada

**Jonathan Huefner**

USA

**Libby Hughes**

Australia

**Nathan Hughes**

Australia

**Carroll W. Hughes**

USA

**Benjamin Hummelen**

Norway

**Kathryn Humphreys**

USA

**Maria Hussain**

Canada

**Natalie Hyde**

Australia

**Benjamin Iffland**

Germany

**Paul Ingram**

USA

**Saleem Iqbal**

Malaysia

**Irmansyah Irmansyah**

Indonesia

**Dorothea Isele**

Germany

**Makoto Ishitobi**

Japan

**Zahinoor Ismail**

Canada

**Matti Isohanni**

Finland

**Tord Ivarsson**

Norway

**Matthias Jaeger**

Switzerland

**Jeremy Jamieson**

USA

**Jens Einar Jansen**

Denmark

**Willemijn Jansen**

The Netherlands

**Robin Jarrett**

USA

**Tariq Javed**

Pakistan

**Dinuk Jayasuriya**

Australia

**Rachel Jenkins**

UK

**Pei Jiang**

China

**Matti Joensuu**

Finland

**David Jolley**

UK

**Erik Jönsson**

Sweden

**Andreas Joos**

Germany

**Matt Judah**

USA

**Minyoung Jung**

South Korea

**Jon Jureidini**

Australia

**Hiroshi Kadotani**

Japan

**Michael Kaess**

Germany

**Astrid Kamperman**

The Netherlands

**Rosalie Kane**

USA

**Daehee Kang**

South Korea

**Yukiko Kano**

Japan

**Joshua Kantrowitz**

USA

**Joshua Kantrowitz**

USA

**Nestor Damian Kapusta**

Austria

**Max Karukivi**

Finland

**Andrea Kass**

USA

**Bracha Katz**

Israel

**Denise Kay**

USA

**Evaldas Kazlauskas**

Lithuania

**Sarah Keedy**

USA

**Jared Keeley**

USA

**Shona Kelly**

UK

**Friederike Kendel**

Germany

**Gary Kennedy**

The Netherlands

**Harry Kennedy**

Ireland

**Zhang Kerang**

China

**Ronald Kessler**

USA

**Mohammadrasoul Khalkhali**

Iran

**Farideh Khodabandeh**

Iran

**Toshiaki Kikuchi**

Japan

**Helen Killaspy**

UK

**David Kilpatrick**

Australia

**Myoung-Hee Kim**

South Korea

**June H. Kim**

USA

**David Kimhy**

USA

**Kylie King**

Australia

**James Kirby**

Australia

**Bonnie Kirsh**

Canada

**Piyanee Klainin-Yobas**

Singapore

**Evan Kleiman**

USA

**Christoph Klein**

UK

**Sarah Knowles**

UK

**Anke Köbach**

Germany

**Michael Koelch**

Germany

**Manja Koenders**

The Netherlands

**Alexandra Koenig**

Germany

**Philip Koester**

Germany

**Sina Kohl**

Germany

**Sebastian Köhler**

The Netherlands

**Parashar Koirala**

Nepal

**Michiko Koizumi**

Japan

**Iris-Tatjana Kolassa**

Germany

**Hristina Koleva**

Uruguay

**Glenn Konopaske**

USA

**Jyrki Korkeila**

Finland

**Lisa Korsbek**

Denmark

**Nikolaos Kotsanos**

Greece

**Hanna Kovshoff**

UK

**Neel Koyawala**

USA

**Ahmet Koyuncu**

USA

**Ueli Kramer**

Switzerland

**Georg Kranz**

Austria

**Mette Felbert Kreis**

USA

**Christina Blanner Kristiansen**

Denmark

**Lisbeth Kristiansen**

Sweden

**Levente Kriston**

Germany

**Kurt Kroenke**

USA

**Jesper Krogh**

Denmark

**Jerome Kroll**

USA

**Daryl Kroner**

USA

**Ralf Kudling**

Germany

**Marina Kukla**

USA

**Sophie L’Heureux**

Canada

**Izelle Labuschagne**

Australia

**Malcolm Lader**

UK

**Te-Jen Lai**

Taiwan

**Edmund Lalor**

Ireland

**Dorian Lamis**

USA

**Hsien-Yuan Lane**

Taiwan

**Fabian Lang**

Germany

**Jean-Philippe Langevin**

USA

**Kate Langley**

UK

**Matthew Large**

Australia

**Victor Lasebikan**

Nigeria

**Jason Lavender**

USA

**C. K. Law**

Hong Kong

**Keyne Law**

USA

**Sharon Lawn**

Australia

**Luisa Lazaro**

Spain

**Jocelyn Lebow**

USA

**David Ledgerwood**

USA

**William Lee**

UK

**Falk Leichsenring**

Germany

**Corey Leidenfrost**

USA

**Erwin Lemche**

UK

**Charlotte Lennox**

UK

**Bethany Leonhardt**

USA

**Dorothea Lerman**

USA

**Tristram Lett**

Canada

**Andrew Leuchter**

USA

**Winnie Leung**

USA

**James Levitt**

USA

**Florence Levy**

Australia

**Terry Lewin**

Australia

**Ute Lewitzka**

Germany

**Zongchang Li**

China

**Lingjiang Li**

China

**Xinzhong Li**

UK

**Yanhui Liao**

China

**Belinda Liddell**

Australia

**Lars Lien**

Norway

**Marijn Lijffijt**

USA

**Lisa Lilenfeld**

USA

**Young-Jin Lim**

South Korea

**Chung-Ying Lin**

Taiwan

**Ramon Lindauer**

The Netherlands

**Olavi Lindfors**

Finland

**Xianchen Liu**

USA

**Augusto Llosa**

France

**Jorge Lopez-Castroman**

Spain

**Francisco López-Muñoz**

USA

**Rosario Lopez-Rodriguez**

Spain

**M. Lowe**

USA

**David Lowery**

UK

**Ciyong Lu**

China

**Cheng-Hsien Lu**

Taiwan

**Xiaosun Lu**

USA

**Juan V. Luciano**

Spain

**Crick Lund**

South Africa

**Amanda Lyall**

USA

**Sean Lynch**

UK

**Paul Lysaker**

USA

**Juncheng Lyu**

China

**Anne Maass**

Germany

**Douglas Macdonald**

USA

**Nuria Machin**

UK

**Ian Maidment**

UK

**Stefanie Malan**

South Africa

**Sarah Manchak**

USA

**Mirko Manchia**

Canada

**Laura Mandelli**

Italy

**Anna Manelis**

USA

**Andrea Mann**

USA

**Kristoffer Månsson**

Sweden

**Lucia Margari**

Italy

**Latisha Marshall**

USA

**Averria Martin**

USA

**Steven Marwaha**

UK

**Yuki Matsumoto**

Japan

**Kayako Matsuo**

Japan

**Naomi Matsuura**

Japan

**Donovan Maust**

USA

**Alexis May**

Canada

**Michel Maziade**

Canada

**Sue Mcandrew**

UK

**Terence Mccann**

Australia

**Michael Mccarthy**

USA

**Alexander Mcgirr**

Canada

**Rebecca Mcketin**

Australia

**Iain Mckinnon**

UK

**Carmen Mclean**

USA

**Gary Mclean**

UK

**Hamish Mcleod**

UK

**Caitlin Mcomish**

Australia

**Graham Meadows**

Australia

**Anja Mehnert**

Germany

**Marco Menchetti**

Italy

**Tito Mendoza**

USA

**Itiana Castro Menezes**

Brazil

**Cecilia Mengo**

USA

**Eva Mennigen**

Germany

**Mahesh Menon**

Canada

**Angela Merkl**

Germany

**David Merrill**

USA

**Verena Metz**

USA

**Magnus Mfoafo-Mccarthy**

Canada

**Jouko Miettunen**

Finland

**Emmanuel Mignot**

USA

**Jeremy Miles**

USA

**Joany Millenaar**

The Netherlands

**Alessandra Minelli**

Italy

**Kyle Minor**

USA

**Regina Miranda**

USA

**Rachel Mitchell**

UK

**Yoshifumi Mizuno**

Japan

**Kristen Moeller-Saxone**

Australia

**Jorge Moncayo Gaete**

Ecuador

**Valeria Mondelli**

UK

**Yukifumi Monden**

Japan

**Joan Monin**

USA

**Michael Montagne**

USA

**Jesus Montero-Marin**

Spain

**Michael Moore**

USA

**Elaine Morrato**

USA

**Derek Morris**

Ireland

**Dominik Moser**

USA

**James Mugisha**

Uganda

**Eimear Muir-Cochrane**

Australia

**Elizabeta Mukaetova-Ladinska**

UK

**Cornelis Mulder**

The Netherlands

**Hendrik Müller**

Germany

**Adrian Mundt**

UK

**Gioia Mura**

Italy

**Stuart Murray**

Australia

**Andrea Murru**

Spain

**Maria Muzik**

USA

**Kathleen Myers**

USA

**Lisa Najavits**

USA

**Takashi Nakamae**

Japan

**Motoaki Nakamura**

Japan

**Kiumars Nasseri**

USA

**Barnabas Natamba**

Uganda

**Carryl Navalta**

USA

**Kim Nazi**

USA

**Bogdan Nemes**

Romania

**Emily Neuhaus**

USA

**Giles Newton-Howes**

New Zealand

**Thinh Nguyen**

Australia

**Angela Nickerson**

Australia

**Kristin Nicodemus**

UK

**René Ernst Nielsen**

Denmark

**Dorien Nieman**

The Netherlands

**Jason Nieuwsma**

USA

**Molly Nikolas**

USA

**Shota Nishitani**

Japan

**Neil Nixon**

UK

**Raquel Nogueira**

Spain

**Axel Nordenskjöld**

Sweden

**William Obrien**

USA

**Stephen O'Connor**

USA

**Brian Odonoghue**

Australia

**Toshiyasu Ogata**

Japan

**Gabriel Oh**

Canada

**Jeneva Ohan**

Australia

**Kazutaka Ohi**

USA

**Takashi Okada**

Japan

**Yasuyuki Okumura**

Japan

**Michelle Okun**

USA

**John Oldham**

USA

**Emilie Olie**

France

**Sepideh Omidvari**

Iran

**Conor O'Neill**

Ireland

**Sian Oram**

UK

**Claire O'Reilly**

Australia

**Katherine Ornstein**

USA

**Marlene Oscar-Berman**

USA

**Theresa Osypuk**

USA

**Caleb Othieno**

Kenya

**Dennis Ougrin**

UK

**Richard Owen**

USA

**Shiro Ozasa**

Japan

**Yuji Ozeki**

Japan

**Cahit Ozer**

Turkey

**Andrew Page**

Australia

**Nagesh Pai**

Australia

**Amir Pakpour**

Iran

**Wang Yuan Pang**

Brazil

**Joel Paris**

Canada

**Gordon Parker**

Australia

**Stephen Parker**

Australia

**Natalie Parletta**

Australia

**Suravi Patra**

India

**Nalin Payakachat**

USA

**Lars Henning Pedersen**

Denmark

**Kirsi Peltonen**

France

**Karl Peltzer**

Thailand

**Sue Penckofer**

USA

**Xie Peng**

China

**Patricia Penn**

USA

**David Perez**

USA

**Christopher Perlman**

Canada

**Giulio Perugi**

Italy

**John Peteet**

USA

**Inge Petersen**

South Africa

**Geraldine Petit**

Belgium

**Melissa Petrakis**

Germany

**Noah Philip**

USA

**Michael Phillips**

China

**Joseph Pierre**

USA

**Aravind Pillai**

USA

**Brian Piper**

USA

**Paul L. Plener**

Germany

**Martin Plöderl**

Austria

**Guilherme Polanczyk**

Brazil

**Elisabetta Poluzzi**

Italy

**Nunzio Pomara**

USA

**Alfredo Pompili**

Italy

**Maurizio Pompili**

Italy

**Raffaele Popolo**

Italy

**V. Porredi**

India

**Richard Porter**

New Zealand

**Vita Postuvan**

Slovenia

**Robert Powers**

USA

**Konasale Prasad**

USA

**Nicole Praschak-Rieder**

Austria

**Antonio Preti**

Italy

**Emanuele Preti**

Italy

**Marijn Alice Prins**

The Netherlands

**Seth Prins**

USA

**James J. Prisciandaro**

USA

**Laura Pryor**

Canada

**Eli Puterman**

USA

**Zhiyong Qu**

China

**Shae E. Quirk**

Australia

**Caroline Quoilin**

Belgium

**R. Radakovic**

UK

**Thilini Rajapakse**

Sri Lanka

**Inez Ramakers**

The Netherlands

**Patti Ranahan**

Canada

**Henrik Rasmussen**

Denmark

**Sujit Rathod**

UK

**Sheila Rauch**

USA

**Lauren Rayner**

UK

**Nicola Reavley**

Australia

**P. Regmi**

UK

**Lennart Reifels**

Australia

**Laoise Renwick**

UK

**János Réthelyi**

Hungary

**Mae Lynn Reyes-Rodriguez**

USA

**Penny Rhodes**

UK

**Wagner Ribeiro**

UK

**Valdo Ricca**

Italy

**Marnie Rice**

Canada

**Frances Rice**

UK

**Derek Richards**

Ireland

**Dieter Riemann**

Germany

**Renee Rienecke**

USA

**Daniela Di Riso**

Italy

**Jesus Rivera-Navarro**

Spain

**Laurence Robel**

France

**Bayard Roberts**

UK

**Eduardo Rocha**

Brazil

**Eric Roche**

Ireland

**Alcibiades Rodriguez**

USA

**Cathryn Rodway**

UK

**David Roe**

Israel

**Darrin Rogers**

USA

**Eline Borger Rognli**

Norway

**Matthias Romppel**

Germany

**Kathlyn Ronaldson**

Australia

**Lucy B Rorke-Adams**

USA

**Laura Rosella**

Canada

**David Rosmarin**

USA

**Francesco Rotella**

Italy

**Dietrich Rothenbacher**

Germany

**Benoit Roussel**

France

**Michael Roy**

USA

**Jorun Rugkåsa**

Norway

**Dan Rujescu**

Germany

**Corrine Ruktanonchai**

USA

**Christine Rummel-Kluge**

Germany

**Anthony Ruocco**

Canada

**Torleif Ruud**

Norway

**Joanne Ryan**

Australia

**Joseph Ryan**

USA

**Natalie Sabik**

Romania

**Sarven Sabunciyan**

USA

**Paul Sacco**

USA

**Naomi Sadeh**

USA

**Anne Sadler**

USA

**Cheryl Sadowski**

Canada

**Tolulope Sajobi**

Canada

**Amy Lynn Salisbury**

USA

**Virginio Salvi**

Italy

**Mark Salzer**

USA

**Federica Sancassiani**

Italy

**Paul Sandor**

Canada

**Paul Sandor**

USA

**Gabriella Santangelo**

Italy

**Michael Saraga**

Switzerland

**Marco Sarchiapone**

Italy

**Ulrik Sartipy**

Sweden

**Alexander Sartorius**

Germany

**Tsuyoshi Sasaki**

Japan

**Heribert Sattel**

Germany

**Emily Saurman**

Australia

**iovanni Savettieri**

Italy

**Assanangkornchai Sawitri**

Thailand

**Ingo Schäfer**

Germany

**Ayal Schaffer**

Canada

**Inga Schalinski**

Germany

**Carlos Schenk**

USA

**Jeffrey Scherrer**

USA

**Ben Schmand**

The Netherlands

**Pedro Antônio Schmidt Do Prado-Lima**

Brazil

**Emilie Schmits**

Belgium

**Anna Schneider**

Germany

**Peter Schofield**

Australia

**Casey Schofield**

USA

**Hannah Schreier**

USA

**Emma Sciberras**

Australia

**Whitney Scott**

UK

**Lisa Segre**

USA

**Chris Segrin**

USA

**Cristina Segura-Garcia**

Italy

**Cl Seifert**

Germany

**Bill Sellwood**

UK

**Sudarshi Seneviratne**

Sri Lanka

**Ömer Senormanci**

Turkey

**Allan Seppanen**

Finland

**Lucy Serpell**

UK

**Antoni Serrano**

Spain

**Michael Seto**

Canada

**Alexander Shackman**

USA

**Asim Shah**

USA

**Amanda Shallcross**

USA

**Katherine Sharkey**

USA

**Rajiv Sharma**

USA

**Jenny Shaw**

UK

**Lindsay Shea**

USA

**Taiwo Lateef Sheikh**

Nigeria

**Yuyan Shi**

USA

**Pedro Shiozawa**

Brazil

**Martha Shumway**

USA

**Yao Shuqiao**

China

**Tianmei Si**

China

**Cecilia Sighinolfi**

Italy

**Greg Simon**

USA

**Panagiotis Simos**

Greece

**Emma Simpson**

UK

**Fatma Simsek**

Turkey

**Duncan Sinclair**

Australia

**Susanne Singer**

Germany

**Meghna Singhal**

India

**Daisy Singla**

USA

**Mark Sinyor**

Canada

**Dan Siskind**

Australia

**Thiagarajan (Raj) Sitharthan**

Australia

**Bram Sizoo**

The Netherlands

**Petros Skapinakis**

Greece

**Gudmundur Skarphedinsson**

Norway

**Silje Skrede**

Norway

**Alexander Slade**

USA

**Matthew Smith**

USA

**Niels Smits**

The Netherlands

**Antigonos Sochos**

UK

**Charlotte Sonne**

Denmark

**Tobias Sonntag**

Germany

**Renan Souza**

Brazil

**Gianfranco Spalletta**

Italy

**William Spaulding**

USA

**Jeffrey Spielberg**

USA

**Alessio Squassina**

Italy

**Jospeh Ssenyonga**

Uganda

**Shauna Stahlman**

USA

**Justine Stang**

Germany

**Stephen Stansfeld**

UK

**Robert Stanton**

Australia

**Vesta Steibliene**

Lithuania

**Dejan Stevanovic**

Serbia And Montenegro

**Donna Stewart**

Canada

**Robert Stewart**

Malawi

**Jeremy Stewart**

USA

**Justin Storbeck**

USA

**Eric Strachan**

USA

**Jeffrey Strawn**

USA

**David Streiner**

Canada

**aniel Strunk**

USA

**Brendon Stubbs**

UK

**Ruibin Su**

China

**Mythily Subramaniam**

Singapore

**Yehuan Sun**

China

**e-Huan Sun**

China

**Long Sun**

China

**Xiang Sun**

UK

**Ye-Huan Sun**

China

**Craig Surman**

USA

**Jaana Suvisaari**

Finland

**Hanako Suzuki**

Japan

**Elisabeth Svensson**

Sweden

**Susan Swider**

USA

**Jennifer Syvertsen**

USA

**Mariana Szklo-Coxe**

USA

**Kota Takaoka**

Canada

**Ardesheer Talati**

USA

**Jody Tanabe**

USA

**Jennifer Yee-Man Tang**

Hong Kong

**Pekka Tani**

Finland

**Cumhur Tas**

Turkey

**Alvin Kuowei Tay**

Australia

**Thomas W Teasdale**

Denmark

**Helio Teive**

Brazil

**Ghazel Tellawi**

USA

**Freek Ten Doesschate**

The Netherlands

**Elena Tenconi**

Italy

**Sampath Udaya Bandara Tennakoon**

Sri Lanka

**Berend Terluin**

The Netherlands

**Fabian Termorshuizen**

The Netherlands

**A Terrill**

USA

**Markos Tesfaye**

Ethiopia

**Christian Thielscher**

Germany

**Neil Thomas**

Australia

**Rae Thomas**

Australia

**Barbara Thomlison**

USA

**Jim Thompson**

Cambodia

**Arun Tiwari**

Canada

**Christian Tjagvad**

Norway

**Weitse Tol**

Germany

**Magdalena Tolea**

USA

**Carmine Tomasetti**

Italy

**Cecilia Tomori**

USA

**Stefania Toselli**

Italy

**Sheri Towe**

USA

**Quynh Anh Tran**

Vietnam

**Sebastian Trautmann**

Germany

**Joel Tremblay**

Canada

**Jonathan Tritter**

UK

**Jack Tsai**

USA

**Matthew Tull**

USA

**Alyna Turner**

Australia

**Barbara J Turner**

USA

**Carolyn Turvey**

USA

**Massimo Tusconi**

Italy

**Hiroyuki Uchida**

Japan

**Peter Uhlhaas**

UK

**D Ukueberuwa**

USA

**Lisa Underwood**

New Zealand

**Rachel Upthegrove**

UK

**Masahide Usami**

Japan

**David Valentiner**

USA

**Wouter Van Ballegooijen**

The Netherlands

**Diana Van Bergen**

The Netherlands

**Rutger Jan Van Der Gaag**

The Netherlands

**Saskia Van Der Oord**

Belgium

**Neeltje Van Haren**

The Netherlands

**Tamsyn Van Rheenen**

Australia

**Eeske Van Roekel**

The Netherlands

**Yvonne Vanneste**

The Netherlands

**M. Varga**

USA

**Marlos Vasconcelos Rocha**

Brazil

**Michael Vaughn**

USA

**Juliana Vaz**

Brazil

**Sarah Victor**

Canada

**Simone Vigod**

Canada

**Roger Vilardaga**

USA

**Anna Vinkhuyzen**

Australia

**Tua Vinther-Jensen**

Denmark

**Jen Vohs**

USA

**Jan Volavka**

USA

**Silke Von Esenwein**

USA

**Benjamin Vyssoki**

Austria

**Elaine Waddington**

Canada

**Tracey Wade**

Australia

**Aurelia Wagener**

Belgium

**Gwenyth Wallen**

USA

**Elizabeth Walsh**

Ireland

**Yiming Wang**

China

**Liang-Jen Wang**

Taiwan

**Peng-Wei Wang**

Taiwan

**Wei Wang**

China

**Klaas Wardenaar**

The Netherlands

**Jason Washburn**

USA

**Flavie Waters**

Australia

**Amy Watson**

USA

**Jessica Weafer**

USA

**Addie Weaver**

USA

**Cesar Weber**

Brazil

**Roland Weierstall**

Germany

**Anthony Weiss**

USA

**Tony Wells**

USA

**Benedict Weobong**

UK

**Nomi Werbeloff**

UK

**P. Michiel Westenberg**

The Netherlands

**Reagan Wetherill**

USA

**Thomas Wetter**

Germany

**Emily White**

USA

**Julie Whitney**

UK

**Richard Whittington**

UK

**Cynthia Wible**

USA

**Beth Wildman**

USA

**Nicola Wiles**

UK

**Sarah Wilker**

Germany

**Joanne Williams**

Australia

**Anna Williamson**

Australia

**Yk Wing**

Hong Kong

**Hans-Ulrich Wittchen**

Germany

**Cilia Witteman**

The Netherlands

**Miranda Wolpert**

UK

**Friedrich Wurst**

Germany

**Til Wykes**

UK

**Yong Xu**

China

**Wang Xueyi**

China

**Joel Yager**

USA

**Atsurou Yamada**

Japan

**Chunsong Yang**

China

**Philip Yanos**

USA

**Xu Yi**

China

**Yuan Yong Gui**

China

**Ki-Bong Yoo**

South Korea

**Jong Yoon**

USA

**Yonggui Yuan**

China

**Xiaojun Yuan**

USA

**David Yusko**

USA

**Renzo Zanotti**

Italy

**Phyllis Zelkowitz**

Canada

**Cristian Zeni**

USA

**Xiang Rong Zhang**

China

**Jie Zhang**

USA

**Yuying Zhang**

China

**Tianhong Zhang**

China

**Min Zhao**

China

**Naiqing Zhao**

China

**Zhi Zheng**

USA

**Qiuyue Zhong**

USA

**Yaara Zisman-Ilani**

USA

**Julie Zito**

USA

**Lisette Zuurbier**

The Netherlands

